# RETRACTION: Inhibition of JNK Alleviates Chronic Hypoperfusion‐Related Ischemia Induces Oxidative Stress and Brain Degeneration via Nrf2/HO‐1 and NF‐κB Signaling

**DOI:** 10.1155/omcl/9789312

**Published:** 2026-06-23

**Authors:** Oxidative Medicine and Cellular Longevity

RETRACTION: M. S. Khan, A. Khan, S. Ahmad, et al., “Inhibition of JNK Alleviates Chronic Hypoperfusion‐Related Ischemia Induces Oxidative Stress and Brain Degeneration via Nrf2/HO‐1 and NF‐κB Signaling,” *Oxidative Medicine and Cellular Longevity* (2020): e5291852, https://doi.org/10.1155/2020/5291852.

The above article, published online on 16 June 2020 in Wiley Online Library (wileyonlinelibrary.com), has been retracted by agreement between the journal Editor‐in‐Chief, Jeannette Vasquez Vivar; and Wiley Periodicals LLC. Concerns were raised by Elizabeth Bik on PubPeer [1] that the control panel in Figure 5B and parts of isch + SP in Figure 3F overlap. The isch + SP panel of Figure 5B also appears to be identical to the control panel found in Figure 2J of a previously published article by members of the same author group [2]. Additionally, an article published in 2021 by some of the same authors includes a duplicate version of this article’s Figure 3D [3]. The publisher confirmed these concerns. The authors responded by providing their raw image data, but their responses to the concerns regarding image duplication were insufficient. Because the images appear to be manipulated, the Editors have lost confidence in the results published. As a result, the data and conclusions are considered unreliable. Therefore, the article must be retracted.
